# Investigation of antiviral state mediated by interferon-inducible transmembrane protein 1 induced by H9N2 virus and inactivated viral particle in human endothelial cells

**DOI:** 10.1186/s12985-017-0875-5

**Published:** 2017-11-03

**Authors:** Bo Feng, Lihong Zhao, Wei Wang, Jianfang Wang, Hongyan Wang, Huiqin Duan, Jianjun Zhang, Jian Qiao

**Affiliations:** 1 Department of Pathophysiology, College of Veterinary Medicine, China Agricultural University, Beijing, 100193 People’s Republic of China; 2 Department of Microbiology and Immunology, Shanxi Medical University, Taiyuan, 030001 Shanxi People’s Republic of China; 3 Beijing Key Laboratory of Traditional Chinese Veterinary Medicine, Beijing University of Agriculture, Beijing, 102206 People’s Republic of China

**Keywords:** H9N2 influenza virus, Inactivated viral particle, IFITM1, Antiviral state, Human endothelial cells, Human epithelial cells

## Abstract

**Background:**

Endothelial cells are believed to play an important role in response to virus infection. Our previous microarray analysis showed that H9N2 virus infection and inactivated viral particle inoculation increased the expression of interferon-inducible transmembrane protein 1 (IFITM1) in human umbilical vein endothelial cells (HUVECs). In present study, we deeply investigated the expression patterns of IFITM1 and IFITM1-mediated antiviral response induced by H9N2 virus infection and inactivated viral particle inoculation in HUVECs. Epithelial cells that are considered target cells of the influenza virus were selected as a reference control.

**Methods:**

First, we quantified the expression levels of IFITM1 in HUVECs induced by H9N2 virus infection or viral particle inoculation using quantitative real-time PCR and western blot. Second, we observed whether hemagglutinin or neuraminidase affected IFITM1 expression in HUVECs. Finally, we investigated the effect of induced-IFITM1 on the antiviral state in HUVECs by siRNA and activation plasmid transfection.

**Results:**

Both H9N2 virus infection and viral particle inoculation increased the expression of IFITM1 without elevating the levels of interferon-ɑ/β in HUVECs. HA or NA protein binding alone is not sufficient to increase the levels of IFITM1 and interferon-ɑ/β in HUVECs. IFITM1 induced by viral particle inoculation significantly decreased the virus titers in culture supernatants of HUVECs.

**Conclusions:**

Our results showed that inactivated viral particle inoculation increased the expression of IFITM1 at mRNA and protein levels. Moreover, the induction of IFITM1 expression mediated the antiviral state in HUVECs.

## Background

H9N2 influenza virus exists on all continents except Antarctica and is the most common subtype of influenza viruses isolated from poultry (chickens and ducks) in China [1, 2]. H9N2 viruses have been isolated from pigs and humans with influenza-like illness in Hong Kong and Mainland China [3–9], demonstrating that the H9N2 influenza virus could cross species barriers and expand its host range from birds to mammalians. Recently, a research showed that H9N2 viruses and pandemic H1N1 viruses have high genetic compatibility and they can produce higher pathogenic reassortment in experimental condition [10]. In addition, H9N2 viruses provide their six inner genes to contribute to the evolution of the H7N9, H10N8 and H5N6 viruses that cause severe human respiratory infections in China [11–13]. All these features indicate that H9N2 virus has a considerable public health threat. Thus, it is valuable to reveal the pathogenesis of H9N2 influenza virus infection and the innate immune responses of host to the H9N2 viruses.

Pathogen invasion could be recognized by pattern recognition receptors (PRRs) and results in production of interferon [14]. The type I interferon binds to interferon-α/β receptors and activates JAK-STAT signaling pathway, resulting in the expression of hundreds of interferon-stimulated genes (ISGs) [15]. Products of these genes are mostly antiviral proteins which are essential for mediating the antiviral state of the host. Interferon-induced transmembrane proteins (IFITMs) were identified nearly 30 years ago, and although other functions have been proposed, the primary role of IFITM proteins seems to be antiviral. IFITM proteins are a family of small transmembrane proteins that are induced strongly by interferon, but that are also expressed basally in several cell types and lines [16, 17]. To date, three IFITM proteins (IFITM1, IFITM2 and IFITM3) have been identified to display broad spectrum antiviral activity in human and mice [18, 19]. Especially, the IFITM1 and IFITM3 were proven to be more effective in resisting influenza virus infection [20, 21]. The siRNA-mediated depletion of IFITMs significantly increased H1N1 virus titers in primary lung fibroblasts and in HeLa cells, while overexpression of IFITM1, 2, 3 could improve resistance to the H3N2 influenza in A549 and MDCK cell lines [22]. Although the antiviral activity of IFITMs has been identified in multiple types of cells, the antiviral activity of IFITM1 has not been reported in endothelial cells infected with H9N2 virus or inoculated with inactivated viral particle.

Generally, airway epithelial cells are considered to be the main target cells of influenza viruses because of expressing two kinds of influenza virus receptors (α-2, 6- and α-2, 3-linked sialic acid receptors) [23–25]. But the more and more evidences indicated that endothelial cells might play an important role in response to influenza virus infection. Most endothelial cells are also showed to express two kinds of influenza virus receptors which provide potential possibility for influenza virus infection in endothelial cells [26]. H5N1 and H7N9 influenza viruses have been proven to directly infect human lung microvascular endothelial cells and replicate in endothelial cell lines [27, 28]. Several studies indicated that endothelial cells might be the source of the cytokines and involved in lung injury during influenza virus infection [29–32]. So, it is important to understand the cellular responses in endothelial cell during influenza virus infection. Our previous study revealed that H9N2 virus could infect human umbilical vein endothelial cells (HUVECs) and induce high level expression of several ISGs, especially IFITM1 [33]. The microarray results showed that both H9N2 virus infection and inactivated viral particle inoculation increase the expression of IFITM1 at transcription level, and the viral particle inoculation induces a higher level (44.75 folds) of IFITM1 than that (8.53 folds) induced by virus infection. However, the level of IFITM1 needs to be quantified by Real-Time PCR and western blot, and little is known about the antiviral activity of IFITM1 induced by H9N2 virus and viral particle in HUVECs. The present study aimed to quantify the expression of IFITM1 induced by H9N2 virus and viral particle and investigate the antiviral state mediated by IFITM1 in HUVECs. And the results showed that both H9N2 virus infection and viral particle inoculation significantly increased the expression of IFITM1 at mRNA and protein levels, and the IFITM1 protein induced by viral particle inoculation significantly enhanced the antiviral state of HUVECs against H9N2 virus infection.

## Methods

### Experiment protocol

We first quantified the expression levels of IFITM1 in HUVECs induced by H9N2 virus infection or viral particle inoculation using quantitative real-time PCR and western blot. Secondly, we detected levels of interferon-ɑ/β using ELISA kits. Thirdly, we observed the effect of HA and NA on IFITM1 expression, and located the position of inactivated viral particles in HUVECs. Finally, we investigated IFITM1-mediated antiviral response by siRNA and activation plasmid transfection. To compare the expression patterns of IFITM1 between endothelial cells and epithelial cells, all of the above experiments were performed on human epithelial cells at the same time.

### Cells culture

Human umbilical vein endothelial cells (HUVECs, CRL-1730, ATCC), human bronchus epithelial cells (BEAS-2Bs, CRL-9609, ATCC) were used for detecting the expression of IFITM1 and secretion of interferon-ɑ/β. Madin Darby canine kidney cells (MDCK, CCL-34, ATCC) were used for plaque assay. These cell lines were cultured in DMEM (Gibco) supplemented with 10% FBS (Gibco), 100 U/mL of penicillin G and 100 μg/mL of streptomycin (Gibco) at 37 °C in a 5% CO_2_ incubator. The cells in passages 5~8 were seeded into six-well plates and cultured for 24 h before each experiment.

### Virus, virus infection and virus inactivation

The H9N2 virus used in this study was A/Chicken/Hebei/4/2008 (H9N2) (Ck/HB/4/08). The complete genome sequences of the virus are available from GenBank under accession numbers FJ499463–FJ49947033. The viruses were propagated in 9-day-old embryonated eggs from specific-pathogen-free (SPF) hens at 37 °C for 60 to 72 h. Virus titers were determined by plaque assay. Our previous study demonstrated that exogenous trypsin was required for the efficient replication of the H9N2 virus in HUVECs [33]. Thus, 0.25 μg/mL exogenousl-1-tosylamide-2-phenylethyl chloromethyl ketone (TPCK)-treated trypsin was added to the medium for all experiments. To inspect the interaction between viral particles and HUVECs, we created inactivated H9N2 viral particles by using 0.094% β-propionolactone (SERVA Electrophoresis) according to previous description [34], and plaque assay was used to evaluate whether viral replicative capacity was completely destroyed.

### Quantitative real-time PCR analysis

To quantify the levels of IFITM1 induced by H9N2 virus and viral particle, HUVECs or BEAS-2Bs were divided into three groups: control group (inoculation with virus-free media), H9N2 virus group (infection with virus at a multiplicity of infection (MOI) of 5) and viral particle group (inoculation with viral particles at a MOI of 5). To detect the effect of HA and NA (Sino Biological) on the mRNA expression of IFITM1, HUVECs or BEAS-2Bs were divided into control group, low concentration group, high concentration group. Cells in control group were inoculated with free media, cells in low and high concentration groups were respectively treated with 0.1 μg/mL and 1 μg/mL HA or NA [35, 36]. RNA in each group used for Real-Time PCR analysis was extracted using TRIzol reagent (Invitrogen) at 6, 12 and 24 h after treatment. cDNA was synthesized using random hexamer and a superscript III reverse transcriptase kit (Invitrogen). Real-time PCR was performed on the cDNA for RNA quantitation using Ex TaqMix (Takara Bio) and Eva green (Biotium) for IFITM1. GAPDH was amplified in parallel with the target genes as an endogenous control and all samples were analyzed in triplicate. The fold changes of specific mRNA from different groups compared to control group. The primer sequences were used as follows: GAPDH, F (5′ to 3′): ACAACTTT GGTATCGTGGAAGGAC and R (5′ to 3′): AGGGATGATGTTCTGGAGAGCC, IFITM1, F (5′ to 3′): ACTGAAACGACAGGGGAAAG and R (5′ to 3′): GAACAGGGACCAGACGACAT.

### Western blot analysis

Cells in each group used for western blot analysis were collected at 12, 24 36 h after treatment and lysed with RIPA lysis buffer (Cell Signaling Technology). The cell lysates were centrifuged and the resultant supernatants were resolved by SDS-polyacrylamide gel electrophoresis (SDS-PAGE) and then transferred onto polyvinylidene difluoride (PVDF) membranes (Roche). The membranes were blocked with 5% skim milk and probed with a monoclonal antibody to β-actin (Santa Cruz Biotechnology), IFITM1 (Sigma-Aldrich). After a further incubation with peroxidase (HRP) conjugated secondary antibody (ORIGENE). Proteins were visualized using enhanced chemiluminescence. The relative protein level of IFITM1 to β-actin was analyzed by Image J software.

### ELISA assay

ELISA assay was used to quantify the levels of interferon-ɑ/β. Supernatant in each group was collected at 6, 12 and 24 h postinfection, and analyzed by ELISA kits (R & D Systems). According to previous description [37, 38], cells in positive control group were treated with poly I: C (Sigma) at the concentration of 10 μg/mL.

### Plaque assay

The plaque assay was used to detect the virus titers propagated in eggs and cell culture supernatant. Plaque assay was performed on MDCK cells as described previously [39]. Briefly, MDCK cells were seeded into 6-well plates. Confluent monolayers were washed with phosphate buffered saline (PBS) and infected with H9N2 virus or cell culture supernatants. The inoculum was discarded after incubation for 1 h and the remaining cells were washed with PBS. An overlay consisting of a mixture of 1.6% agarose (Lonza) and double-strength DMEM with 0.25 μg/mL TPCK-trypsin (Worthington) was added to the above cells and incubated at 37 °C for 72 h. Plaques were stained with 0.1% crystal violet and counted.

### siRNA transfection

For interference assay, cells were transfected with control siRNA or IFITM1 specific siRNA using Lipofectamine 3000 transfection reagent kit (Invitrogen). IFITM1 specific and control siRNA used in the study were purchased from Santa Cruz Biotechnology, and the siRNA for human cells is a pool of 3 target-specific 19–25 nt siRNAs designed to knock down gene expression. According to instructions of products, we firstly prepared mixtures (0.2 μM siRNA and 7.5 μL Lipofectamine 3000) of siRNA and Lipofectamine 3000 transfection reagents using Opti-MEM Media. To interfere the expression of IFITM1, cells were infected with H9N2 virus or viral particle and incubated for 1 h, then cells were covered with mixtures for 36 h at 37 °C in a 5% CO_2_ incubator. The interference effect of IFITM1 specific siRNA was detected by western blot at 36 h postinfection.

### IFITM1 CRISPR activation plasmid transfection

For overexpression assay, cells were transfected with control plasmid or IFITM1 CRISPR activation plasmid (Santa Cruz Biotechnology) using Lipofectamine 3000 transfection reagent kit (Invitrogen). The IFITM1 CRISPER activating plasmid is a synergy activation medium (SAM) transcriptional activation system designed to specifically upregulate gene expression. According to instructions of products, we firstly prepared mixtures (2 μg plasmid and 7.5 μL Lipofectamine 3000) of plasmid and Lipofectamine 3000 transfection reagents using Opti-MEM Media. Then cells were covered with the mixtures for 36 h at 37 °C in a 5% CO_2_ incubator. The overexpression level of IFITM1 was detected by western blot at 36 h after transfection.

### Immunofluorescence

Immunofluorescence was used to locate the position of viral particles in cells. According to previous description [40], HUVECs or BEAS-2Bs were directly fixed in 2% paraformaldehyde, permeabilized with 0.5% Triton X-100. After blocking with phosphate-buffered saline (PBS) containing 5% bovine serum albumin (BSA) for 1 h at 37 °C. Cells were labeled with anti-nucleoprotein (NP) antibody A-3 (Aviva Systems Biology) overnight, followed by stained with FITC conjugated anti-rabbit secondary antibody (ORIGENE) for 1 h at 37 °C. The images were captured using OLYMPUS fluorescence microscopy.

### Statistical analysis

The data were expressed as means ± standard deviations (SD). All the statistical tests were performed using GraphPad Prism software (version 6.0). Statistical significance of differences were determined using the Student’s t-test or one-way analysis of variance (ANOVA). *P* < 0.05 was considered statistically significant.

## Results

### H9N2 virus infection and viral particle inoculation increased the expression of IFITM1

**Fig. 1:**
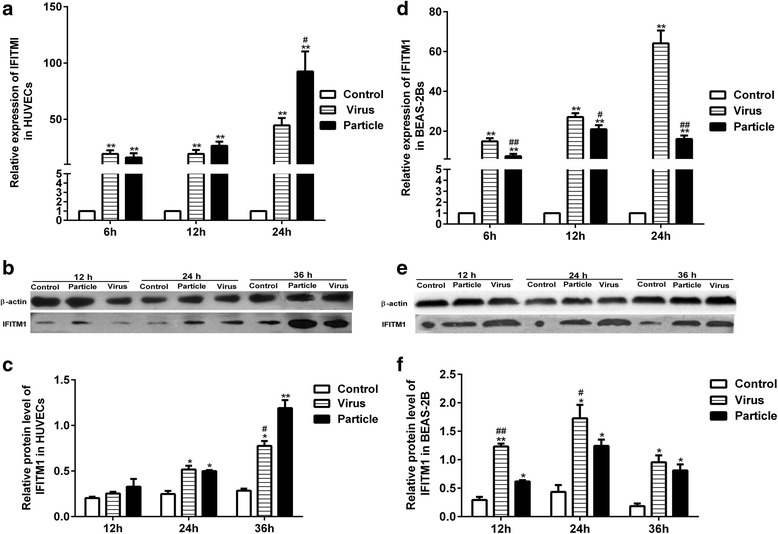
Quantitation of IFITM1 induced by H9N2 virus or inactivated viral particle in HUVECs and BEAS-2Bs. HUVECs and BEAS-2Bs were treated with H9N2 virus (i.e., Virus) or viral particle (i.e., Particle) at MOI of 5. Cells used for RT-PCR and western blot analysis were collected at different time points. **a** The expression of IFITM1 at mRNA level in HUVECs. **b, c** The expression of IFITM1 at protein level in HUVECs, and the relative protein level to β-actin. **d** The expression of IFITM1 at mRNA level in BEAS-2Bs. **e, f** The expression of IFITM1 at protein level in BEAS-2Bs, and the relative protein level to β-actin. * means particle group and virus group compared with control group (*, *P* < 0.05. **, *P* < 0.01, ANOVA). # means particle group compared with H9N2 virus group (#, *P* < 0.05. ##, *P* < 0.01, ANOVA)

According to our previous microarray results, both H9N2 virus infection and inactivated viral particle inoculation upregulate the expression of IFITM1 at transcriptional level. Here we used Real-Time PCR and western blot to quantify the levels of IFITM1 induced by virus infection or viral particle inoculation in HUVECs and BEAS-2Bs. Results showed that both H9N2 virus infection and viral particle inoculation increased the expression of IFITM1 at mRNA and protein levels in HUVECs and BEAS-2Bs (Fig. 1). Compared to virus group, viral particle inoculation induced higher levels of IFITM1 at the mRNA level at 24 h and at protein level at 36 h (*P* < 0.05, ANOVA) in HUVECs (Fig. 1a–c). In contrast, H9N2 virus infection induced higher expression of IFITM1 at mRNA level at 6 h, 12 h, 24 h (*P* < 0.05, ANOVA), and higher levels of IFITM1 protein at 6 h, 12 h (*P* < 0.05, ANOVA) in BEAS-2Bs (Fig. 1d–f). Our data showed that H9N2 virus infection and viral particle inoculation induced different kinetics of IFITM1 expression in/between HUVECs and BEAS-2Bs.

### Levels of interferon-α/β induced by H9N2 virus infection and viral particle inoculation

**Fig. 2:**
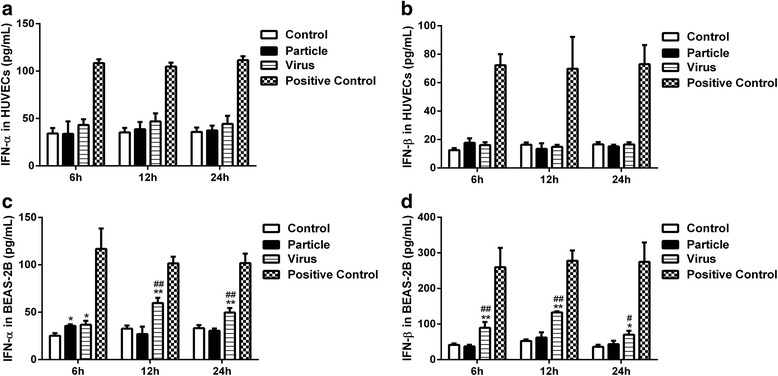
Production of interferon-α/β by HUVECs and BEAS-2Bs inoculated with H9N2 virus or viral particle. HUVECs and BEAS-2Bs were inoculated with H9N2 virus or viral particle at a MOI of 5, and then supernatants were collected at 6, 12, 24 h. Levels of interferon-α/β were detected using ELISA kits. Values represent the means from three independent experiments plus standard deviations. **a, b** The concentration of interferon-α/β in culture supernatants of HUVECs. **c, d** The concentration of interferon-α/β in culture supernatants of BEAS-2Bs. *means particle group and virus group compared with control group (*, *P* < 0.05. **, *P* < 0.01, ANOVA). # means particle group compared with H9N2 virus group (#, *P* < 0.05. ##, *P* < 0.01, ANOVA)

According to previously described, expression of IFITM proteins are potently induced by type I interferon [41]. To determine whether interferon-α/β were involved in the expression of IFITM1 induced by H9N2 virus and viral particle, we treated HUVECs and BEAS-2Bs with H9N2 virus or viral particle at a MOI of 5 and incubated for 1 h. Supernatant in each group was collected at 6, 12 and 24 h postinfection for ELISA assay. Results showed that there was no significant difference (*P* > 0.05, ANOVA) between treated and untreated control group at each time point in HUVECs (Fig. 2a, b), suggesting that interferon-α/β did not participate in the induction of IFITM1 in HUVECs. However, different results were observed in BEAS-2Bs. Compare to control group, levels of interferon-α/β were significantly upregulated at 6, 12 and 24 h postinfection (*P* < 0.05, ANOVA), viral particle inoculation just induced a higher level of interferon-α (*P* < 0.05, ANOVA) at 6 h (Fig. 2c, d). In addition, H9N2 virus infection also induced higher levels of interferon-α/β than that induced by viral particle inoculation in BEAS-2Bs (Fig. 2c, d). The results demonstrated that H9N2 virus and viral particle might share different mechanisms in the induction of IFITM1 expression in HUVECs and BEAS-2Bs.

### HA and NA proteins had no effect on the expression of IFITM1

**Fig. 3:**
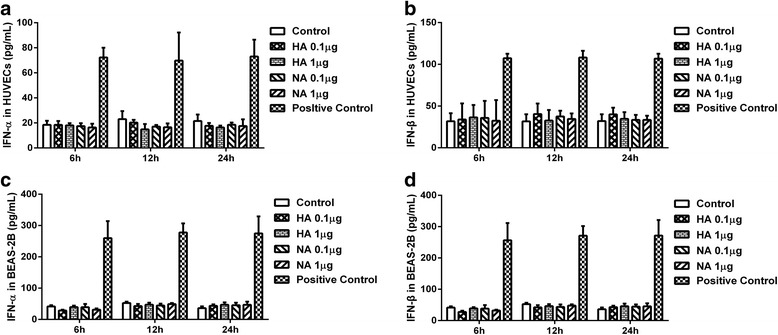
Evaluation of interferon-α/β in HUVECs and BEAS-2B incubated with hemagglutinin (HA) and neuraminidase (NA). HUVECs and BEAS-2Bs were incubated with HA or NA at concentrations of 0.1, 1 μg/mL, supernatants were collected at 6, 12, 24 h. Levels of interferon-α/β were determined using ELISA kits. Values represent the means from three independent experiments plus standard deviations. **a, b** The concentration of interferon-α/β in culture supernatants of HUVECs. **c, d** The concentration of interferon-α/β in culture supernatants of BEAS-2Bs

**Fig. 4:**
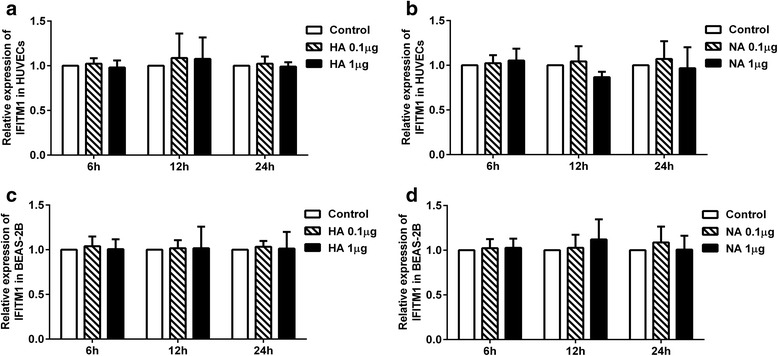
Expression of IFITM1 at mRNA level in HUVECs and BEAS-2Bs treated with hemagglutinin (HA) and neuraminidase (NA). HUVECs and BEAS-2Bs were incubated with HA or NA at concentrations of 0.1, 1 μg/mL. Cells used for RT-PCR analysis were collected at 6, 12, 24 h. **a** The mRNA levels of IFITM1 induced by HA in HUVECs. **b** The mRNA levels of IFITM1 induced by NA in HUVECs. **c** The mRNA levels of IFITM1 induced by HA in BEAS-2Bs. **d** The mRNA levels of IFITM1 induced by NA in BEAS-2Bs

Results in Figs. 1, 2 showed that H9N2 virus infection and viral particle inoculation increased the expression of IFITM1 independently of interferon-α/β in HUVECs. Thus, we investigated whether envelope proteins were involved in the induction of IFITM1 expression. We incubated HUVECs with HA or NA protein at different concentrations. Supernatant in each group was collected at 6, 12 and 24 h for ELISA assay, cells were extracted for Real-Time PCR analysis. The results showed that interferon-α/β levels were not significantly increased after treatment with HA or NA (*P* > 0.05, ANOVA) in both BEAS-2Bs and HUVECs (Fig. 3). As shown in Fig. 4, the mRNA levels of IFITM1 were not significantly increased at 6, 12 and 24 h (*P* > 0.05, ANOVA) in HUVECs and BEAS-2Bs. The results suggested that the HA or NA protein binding alone is not sufficient to induce the expression of IFITM1 in HUVECs or BEAS-2Bs.

### Location of viral particle

**Fig. 5:**
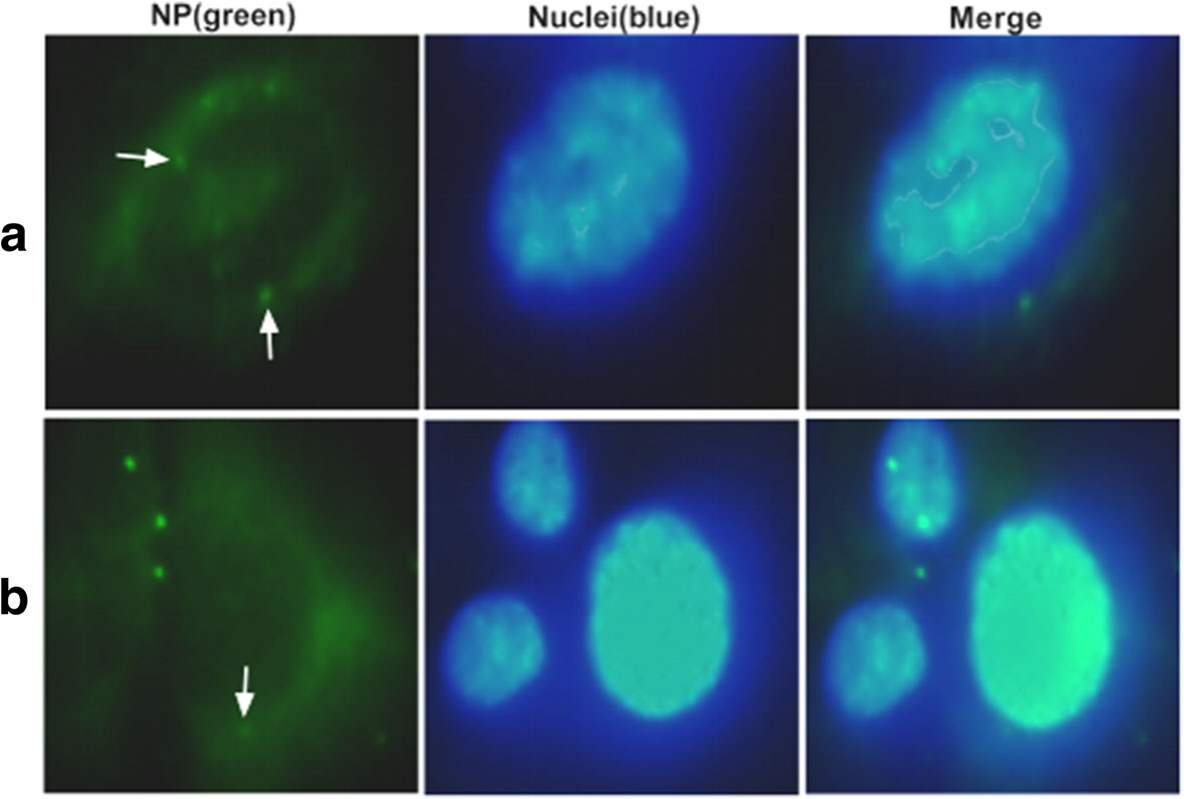
Localization of inactivated viral particles in HUVECs and BEAS-2Bs. HUVECs and BEAS-2Bs were inoculated with viral particles at a MOI of 5 for 8 h, the distribution of viral particles were visualized by immunofluorescence. Cells in each group were double-stained with an anti-nucleoprotein antibody (NP, green) and 4′, 6-diamidino-2-phenylindole (DAPI, blue). **a** Localization of viral particles in HUVECs. **b** Localization of viral particles in BEAS-2Bs

Viral particle inoculation significantly increased the expression of IFITM1 independently of interferon-α/β in HUVECs, and the binding of HA or NA protein alone is not sufficient to increase the IFITM1 level. We suspected that effect of viral particle on IFITM1 expression was generated inside the cells. Then we located viral particles using immunofluorescence. Cells were inoculated with viral particles at a MOI of 5 and incubated for 1 h. According to previous description [27, 42], viral particles were labeled with anti-NP antibody A-3 and FITC conjugated secondary antibody at 8 h after inoculation. Results showed that NP-positive cells were observed after inoculation with viral particles (Fig. 5). The results indicated that the cellular interaction between intracellular molecules and viral particles might be involved in the induction of IFITM1 expression in HUVECs.

### IFITM1 induced by H9N2 virus infection did not significantly enhance the antiviral state

**Fig. 6:**
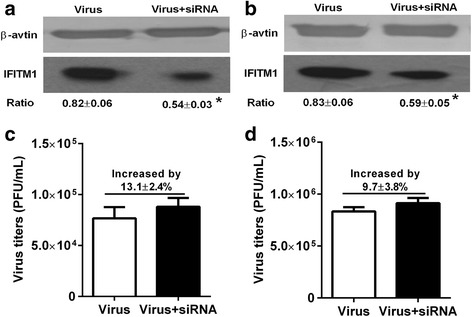
Antiviral activity of IFITM1 induced by H9N2 virus infection in HUVECs and BEAS-2Bs. HUVECs and BEAS-2Bs were infected with H9N2 virus at MOI of 5 and incubated for 1 h, then cells were transfected with control siRNA or IFITM1 specific siRNA for 36 h. Virus titer in each group was detected using plaque assay at 36 h postinfection. The effect of IFIMT1 specific siRNA on IFITM1 expression was detected using western blot. **a** IFITM1 protein level after transfected with siRNA in HUVECs. **b** IFITM1 protein level after transfected with siRNA in BEAS-2Bs. **c** Virus titers in HUVECs transfected with control siRNA or IFITM1 specific siRNA. Compared to virus group (control siRNA), the virus titer in virus + siRNA group (IFITM1 specific siRNA) was increased by 13.1 ± 2.4% (*P* > 0.05, t-test). **d** Virus titers in BEAS-2Bs transfected with control siRNA or IFITM1 specific siRNA. Compared to virus group (control siRNA), the virus titer in virus + siRNA group (specific siRNA) was increased by 9.7% ± 3.8% (*P* > 0.05, t-test)

To determine the antiviral activity of IFITM1 protein induced by H9N2 virus infection, HUVECs or BEAS-2Bs were infected with H9N2 virus at MOI of 5 and incubated for 1 h, then cells were transfected with IFITM1 specific siRNA or control siRNA for 36 h. As shown in Fig. 6a, IFITM1 specific siRNA transfection successfully knocked down the expression of IFITM1 induced by H9N2 virus infection in HUVECs. However, the virus titers were just increased by 13.1 ± 2.4% (*P* > 0.05, t-test) compared with the virus group (control siRNA) (Fig. 6c). Similarly, the expression of IFITM1 induced by H9N2 virus infection was also successfully interfered by IFITM1 specific siRNA transfection in BEAS-2Bs (Fig. 6b). And the virus titers were just increased by 9.7 ± 3.8% (*P* > 0.05, t-test) compared with virus group (control siRNA) (Fig. 6d). The results suggested that IFITM1 induced by H9N2 virus infection did not mediate the antiviral response in HUVECs and BEAS-2Bs.

### IFITM1 induced by viral particle inoculation significantly enhanced the antiviral state

**Fig. 7:**
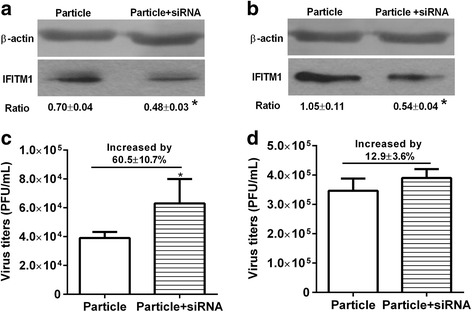
Antiviral activity of IFITM1 induced by viral particle inoculation in HUVECs and BEAS-2Bs. HUVECs and BEAS-2Bs were inoculated with viral particle at MOI of 5 and incubated for 1 h, then cells were transfected with control siRNA or IFITM1 specific siRNA for 36 h before infected with H9N2 virus at MOI of 5. Virus titer of each group was detected by plaque assay at 36 h postinfection. The effect of IFIMT1 specific siRNA on IFITM1 expression was detected using western blot. **a** IFITM1 protein level after transfected with siRNA in HUVECs. **b** IFITM1 protein level after transfected with siRNA in BEAS-2Bs. **c** Virus titers in HUVECs transfected with control siRNA or IFITM1 specific siRNA. Compared to particle group (control siRNA), the virus titer in particle + siRNA group (IFITM1 specific siRNA) was increased by 60.5 ± 10.7% (*P* < 0.05, t-test). **d** Virus titers in BEAS-2Bs transfected with control siRNA or IFITM1 specific siRNA. Compared to particle group (control siRNA), the virus titer in particle + siRNA group (specific siRNA) was increased by 12.9 ± 3.6% (P > 0.05, t-test). * means particle group compared with particle + siRNA group (*, P < 0.05, t-test)

HUVECs or BEAS-2Bs were inoculated with viral particles at MOI of 5 and incubated for 1 h, then cells were transfected with siRNA for 36 h before infected with H9N2 virus. Results showed that siRNA transfection knocked down the expression of IFITM1 induced by viral particle inoculation in HUVECs and BEAS-2Bs (Fig. 7a, b). Compared to particle group, the virus titers were significantly increased by 60.5 ± 10.7% (*P* < 0.05, t-test) after transfection with IFITM1 specific siRNA (Fig. 7c). In contrast, the virus titers were only increased by 12.9 ± 3.6% (*P* > 0.05, t-test) after transfection with IFITM1 specific siRNA in BEAS-2Bs (Fig. 7d). The results indicated that the IFITM1 protein induced by viral particle inoculation significantly enhanced the antiviral state of HUVECs.

### Overexpression of IFITM1 significantly enhance the antiviral state

**Fig. 8:**
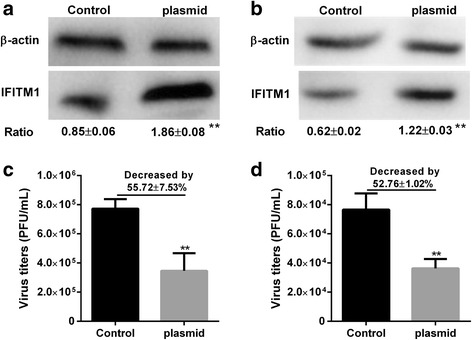
Overexpression of IFITM1 significantly reduced virus titers in HUVECs and BEAS-2Bs. HUVECs and BEAS-2Bs were transfected with control plasmid (Control) or IFITM1 CRISPR activation plasmid (plasmid) for 36 h, then cells were infected with H9N2 virus at MOI of 5. Virus titer of each group was detected by plaque assay at 36 h postinfection. The overexpression of IFIMT1 was detected by western blot. **a** IFITM1 protein level after transfected with plasmid in BEAS-2Bs. **b** IFITM1 protein level after transfected with plasmid in HUVECs. **c** Virus titers in BEAS-2Bs transfected with control plasmid or IFITM1 CRISPR activation plasmid. Compared to control group (control plasmid), the virus titer in plasmid group (IFITM1 CRISPR activation plasmid) was decreased by 55.72.5 ± 7.53% (*P* < 0.01, t-test). **d** Virus titers in HUVECs transfected with control plasmid or IFITM1 CRISPR activation plasmid. Compared to control group (control plasmid), the virus titer in plasmid group (IFITM1 CRISPR activation plasmid) was decreased by 52.76 ± 1.02% (*P* < 0.01, t-test). * means control group compared with plasmid group (**, *P* < 0.01, t-test)

HUVECs or BEAS-2Bs were transfected with plasmid for 36 h before infected with H9N2 virus. The results showed that transfection with IFITM1 CRISPR activation plasmid upregulated the expression of IFITM1 in HUVECs and BEAS-2Bs (Fig. 8a, b). Compared to control group, the virus titers were significantly decreased by 55.72 ± 7.53% (*P* < 0.05, t-test) in BEAS-2Bs transfected with IFITM1 CRISPR activation plasmid (Fig. 8c), the virus titers were significantly decreased by 52.76 ± 1.02% (*P* < 0.05, t-test) in HUVECs transfected with IFITM1 CRISPR activation plasmid (Fig. 8d).

## Discussion

The expression of ISGs is an early response of host to virus infection, and their products confers host the antiviral state which inhibits the entry process or replication of invading virus. To date, multiple proteins translated by ISGs have been validated as antiviral proteins, such as protein kinase R (PKR), myxovirus-resistance proteins, ISG15, Schlafen 11 and so on. IFITM proteins are recently identified antiviral factors that play critical roles in the intrinsic and interferon-mediated control of virus infection [20, 22]. Since initially identified by a siRNA screen for factors that restrict influenza virus replication, more and more researches revealed the inhibition effect of IFITMs on enveloped viruses through affecting the interaction between virus envelope proteins and endosomal or lysosomal [43]. Recently, a study demonstrated that depletion of IFITM1 with siRNA increased titers of H1N1 virus in primary lung fibroblast cells and in HeLa cell line, overexpression of IFITM1 resisted H3N2 virus infection in A549 and MDCK cell lines [22]. In this study, our results showed that overexpression of IFITM1 significantly reduced the virus titers in HUVECs and BEAS-2Bs (Fig. 8). The present data reaffirmed the restriction effect of IFITM1 on influenza virus infection. To investigate whether IFITM1 induced by H9N2 virus infection and viral particle inoculation mediated the antiviral response in HUVECs, we detected the virus titers in HUVECs transfected with IFITM1 specific siRNA or control siRNA. The results showed that IFITM1 induced by H9N2 virus infection could not enhanced the antiviral state in HUVECs. In contrast, IFITM1 induced by viral particle inoculation significantly enhance the antiviral response in HUVECs (Fig. 7). Taking into account the above results, we hypothesized that virus replication preceded the expression of IFITM1 in initially infected cells. A previous study demonstrated that IFITM3 efficiently restricted influenza virus and IFITM1 modestly restricted influenza virus, and the expression patterns of IFITMs are likely to be an independent determinant of viral tropism [20]. Our previous microarray results showed that H9N2 virus and viral particle do not induce the expression of IFITM3 in HUVECs [33]. We speculated that this might be a unique response of HUVECs to H9N2 virus infection or viral particle inoculation. Taken together, our data may offer further insight into the innate immune response of endothelial cells to influenza virus infection.

In the present study, the level of IFITM1 induced by viral particle inoculation was higher than that induced by H9N2 virus infection in HUVECs (Fig. 1). The observation differed to what we generally expected, so it is worthy of thinking the sense of IFITM1 expression upregulated by viral particle. Moreover, a recent study indicated that H9N2 influenza virus infection induces the expression of IFITM1 in lung, heart and liver in BALB/c mice [44]. In vivo, the basal lamina with an average thickness of 1 μm is the only structure which separates epithelial cells and endothelial cells [45]. Death of infected epithelial cells creates gaps and the released virus particles readily access to endothelial cells [46, 47]. Thus, the released virus particles may stimulate the antiviral response in endothelial cells. In addition, cytokines produced by epithelial cells could further activate neighboring endothelial cells during influenza virus infection [48]. So it is conceivable that interferon-α/β released by infected epithelial cells could readily induce the expression of IFITM proteins in endothelial cells. Taken together, we may consider to enhance the antiviral state of host by stimulating the respiratory tract with inactivated viral particles.

Previous studies indicated that H5N1 virus infection up-regulate the expression of type I interferon in human pulmonary microvascular endothelial cells and induce high levels of interferon-β in HUVECs [28, 49]. However, our results showed that expression of IFITM1 induced by H9N2 virus and viral particle independently of interferon-α/β in HUVECs (Fig. 2). Although a recent study demonstrated that human IFITM3 and mouse IFITM3 are induced by cytokines of the gp130 family (such as IL-6) [50], we failed to find a possible mechanism involved in IFITM1 expression. Thus, we investigated the effects of HA and NA proteins on the expression of IFITM1 in HUVECs. The results showed that treatment with HA or NA could not upregulate the levels of interferon-α/β and IFITM1 (Figs. 3, 4). Virus RNA activates the cellular antiviral response has been widely reported. In the present study, we did not investigate the effect of RNA of H9N2 virus on the antiviral response in endothelial cells. However, our results showed that H9N2 virus could infect and replicate in HUVECs with invalid effect on antiviral response of HUVECs. The results indicated that RNA of H9N2 virus may play an insignificant role in the expression of IFITM1 induced by H9N2 virus infection or viral particle inoculation. We speculated that the induction of IFITM1 expression may depend on the interaction between viral particles and cellular factors. Moreover, a previous study demonstrated that interactions between cellular factors and envelope glycoprotein B of replication defective human cytomegalovirus may induce ISGs expression [51]. Then we verified whether the viral particles were taken up by cells using immunofluorescence. The results showed that viral particles could enter the HUVECs (Fig. 5), suggesting that cellular interaction might be involved in the induction of IFITM1 in HUVECs. Obviously, more work needs to be done to explore this induction mechanism, and data of the present study might considerably narrow the range of possible mechanisms.

Generally, human influenza virus and avian influenza virus prefer to infect epithelial cells expressing α-2, 6- and α-2, 3-linked sialic acid receptors [24, 25]. To investigate whether these phenomena were unique responses to H9N2 virus and viral particle in HUVECs, we performed the same experiments on epithelial cells synchronously. Our results showed that endothelial cells and epithelial cells shared different features. Consistent with a previous study [52], H9N2 virus infection and viral particle inoculation elevated the levels of type I interferon in BEAS-2Bs (Fig. 2). In addition, contrary to endothelial cells, H9N2 virus infection induced a higher level of IFITM1 in BEAS-2Bs (Fig. 1). These data suggested that H9N2 virus or viral particle may stimulate the innate immune response via different ways in vivo.

It is of course that further studies are necessary to go beyond our present results. For example, further investigation needs to be done for revealing the precise mechanism of distinct expression patterns of IFITM1 in epithelial cells and endothelial cells. In particular, whether inactivated viral particle inoculation increases IFITM1 expression in endothelial cells in vivo and enhances the antiviral state of host.

## Conclusions

Our results showed that inactivated viral particle inoculation increased the expression of IFITM1 at mRNA and protein levels in HUVECs. Moreover, the induction of IFITM1 expression mediated the antiviral response in HUVECs.

## Abbreviations

BEAS-2Bs: Human bronchus epithelial cells
HA: Hemagglutinin
HUVECs: Human umbilical vein endothelial cells
IFITM1: Interferon-inducible transmembrane proteins 1
MOI: multiplicity of infection
NA: Neuraminidase
